# A Digital Single-Session Intervention (Project Engage) to Address Fear of Negative Evaluation Among College Students: Pilot Randomized Controlled Trial

**DOI:** 10.2196/48926

**Published:** 2023-11-23

**Authors:** Arka Ghosh, Katherine A Cohen, Laura Jans, Carly A Busch, Riley McDanal, Yuanyuan Yang, Katelyn M Cooper, Jessica L Schleider

**Affiliations:** 1 Department of Medical Social Sciences Feinberg School of Medicine Northwestern University Chicago, IL United States; 2 Center for Behavioral Intervention Technologies Feinberg School of Medicine Northwestern University Chicago, IL United States; 3 Research for Inclusive STEM Education Center School of Life Sciences Arizona State University Tempe, AZ United States; 4 Department of Psychology Stony Brook University Stony Brook, NY United States; 5 Department of Psychology University of Kansas Lawrence, KS United States

**Keywords:** single-session intervention, fear of negative evaluation, active learning course, pilot randomized controlled trial, intervention, college student, science course, active learning, negative evaluation

## Abstract

**Background:**

Increasingly, college science courses are transitioning from a traditional lecture format to active learning because students learn more and fail less frequently when they engage in their learning through activities and discussions in class. Fear of negative evaluation (FNE), defined as a student’s sense of dread associated with being unfavorably evaluated while participating in a social situation, discourages undergraduates from participating in small group discussions, whole class discussions, and conversing one-on-one with instructors.

**Objective:**

This study aims to evaluate the acceptability of a novel digital single-session intervention and to assess the feasibility of implementing it in a large enrollment college science course taught in an active learning way.

**Methods:**

To equip undergraduates with skills to cope with FNE and bolster their confidence, clinical psychologists and biology education researchers developed Project Engage, a digital, self-guided single-session intervention for college students. It teaches students strategies for coping with FNE to bolster their confidence. Project Engage provides biologically informed psychoeducation, uses interactive elements for engagement, and helps generate a personalized action plan. We conducted a 2-armed randomized controlled trial to evaluate the acceptability and the preliminary effectiveness of Project Engage compared with an active control condition that provides information on available resources on the college campus.

**Results:**

In a study of 282 upper-level physiology students, participants randomized to complete Project Engage reported a greater increase in overall confidence in engaging in small group discussions (*P*=.01) and whole class discussions (*P*<.001), but not in one-on-one interactions with instructors (*P*=.05), from baseline to immediately after intervention outcomes, compared with participants in an active control condition. Project Engage received a good acceptability rating (1.22 on a scale of –2 to +2) and had a high completion rate (>97%).

**Conclusions:**

This study provides a foundation for a freely available, easily accessible intervention to bolster student confidence for contributing in class.

**Trial Registration:**

OSF Registries osf.io/4ca68 http://osf.io/4ca68

## Introduction

### Overview

For over a decade, national calls have championed the transition of college science courses from the traditional lecture format, where instructors lecture at students who passively listen, to active learning courses, where students engage in their learning during class [1,2]. In active learning courses, students engage in learning by participating in activities, such as clicker questions or worksheets, as well as having discussions with their peers and instructors during class [3]. The national push to adopt active learning resulted from robust evidence suggesting that, on average, students learn more and fail less frequently in active learning science courses than in traditional lecture science courses [4] and demonstrating that active learning narrows the achievement gap in science for students from underrepresented groups [5]. Despite the undeniable benefits of active learning, recent research has found that active learning can cause significant anxiety in undergraduates [6-10]. These feelings of worry and apprehension are primarily due to students’ fears of being negatively evaluated by their classmates and instructors, often instigated by low levels of confidence [6-8]. As a first step toward ameliorating this common student-level challenge, we conducted a randomized pilot evaluation of a novel single-session digital intervention, Project Engage, designed to help students better manage their fear of negative evaluation (FNE) and bolster their confidence within active learning environments.

### Background

FNE is defined as a sense of dread associated with being unfavorably evaluated in a social situation [11,12]. It was first applied in the context of higher education in language-learning courses, where students are regularly expected to engage in discussion during class [13]. However, with the transition of science courses to active learning, there are far more social evaluative situations, or opportunities for students to be negatively judged by their peers, owing to the increased number of conversations during class. Specifically, 2 qualitative interview studies, one focused on students enrolled in large-enrollment active learning college science courses and the other on students enrolled in small-enrollment active learning college science courses, found that students describe that if they contribute their thoughts to a discussion about science and their thoughts are wrong, others will perceive them as *dumb* or *stupid* [6,8]. Students were also worried that a single interaction with their peers or instructor could lead to a lasting negative reputation.

As a result of these feelings of worry and apprehension, undergraduates described that they struggle to think through science problems, have difficulty articulating their thoughts about science in discussions, and avoid participating in conversations [6,8]. A recent study of >500 undergraduates enrolled in large-enrollment active learning science courses echoed these findings; students most commonly reported that FNE caused them to overthink their responses, participate less, struggle to speak, and struggle to think [14]. Undergraduates who are worried about being negatively evaluated may monitor their environment for the threat of potential judgment [15]. This likely increases cognitive load, consequently limiting their ability to think and perform specific tasks [16]. As such, it is unsurprising that this fear of judgment primarily hinders students’ performance in class. In addition, FNE has been linked to low self-confidence [8]. If students’ concerns about being judged in science courses cause them to feel less confident, this may lower their self-efficacy, ultimately negatively affecting their performance [17].

Notably, the solution to these challenges is not to eliminate active learning from college science courses. Overwhelming evidence suggests that reverting to the traditional lecture format would not only be detrimental to student learning but would also be less equitable [4,5]. Further, traditional lectures can also exacerbate feelings of anxiety in students, but for different reasons than active learning courses do [8]. In traditional lectures, students express worry that they are unable to gauge how much they have learned before summative assessments and that there is a lack of opportunities to clarify their understanding with others. Therefore, researchers have suggested a 2-pronged solution to address student FNE in active learning [18]. First, to adjust how active learning practices are implemented, and second, to bolster students’ confidence and ability to cope with the FNE.

In the context of higher education, there have been few documented efforts to reduce social anxiety or bolster confidence in speaking in the classroom. One study of 20 students at the National University of Singapore found that allowing chemistry students to contribute to discussions with partial anonymity (voice only) reduced feelings of anxiety [19]. Another study of >500 business students at a large university found that in classes where instructors frequently cold-called students, defined as calling on students whose hands were not raised, students became more comfortable participating in class, compared with students in classes that did not implement cold-call as frequently [20]. However, no studies have directly targeted FNE in the context of active learning. Reducing FNE is typically considered a goal in the treatment of social anxiety disorder, suggesting that researchers interested in FNE look toward literature in clinical psychology. Intervention strategies from cognitive behavioral therapy (CBT) for social anxiety include providing psychoeducation, encouraging exposure to feared stimuli, discouraging avoidance of feared stimuli, and improving cognitive flexibility [21]. Although there is substantial evidence from meta-analytic studies that CBT-informed treatments are effective, most interventions are intensive, typically requiring between 12 and 16 sessions [22,23]. Such treatment cannot be feasibly implemented for each student engaging in active learning courses. However, there is evidence that single-session interventions (SSIs), defined as “specific, structured programs that intentionally involve just one visit or encounter with a clinic, provider, or program” [24], can result in sustained change.

SSIs are intentionally designed to target clinically relevant mechanisms in a brief, self-contained period. They have been used for a broad array of problems, including anxiety, depression, conduct problems, alcohol abuse, and more [25]. Evidence suggests that these effects are longstanding. For example, individuals randomized to complete a 30-minute, digital, self-administered SSI designed to encourage behavioral activation showed significantly greater decreases in depression at a 3-month follow-up compared with individuals randomized to an active control condition [26]. Across multiple trials, both youths and young adults, who report limited access to traditional mental health services owing to a variety of structural- and stigma-related barriers [27], rated SSIs as highly acceptable [26,28,29].

The reduced burden of SSIs, particularly those that are delivered digitally and are self-administered, allows for their dissemination in a wide variety of settings, including classrooms. Several mental health–focused trials have found positive psychopathological outcomes of SSIs in school settings [30-33], including higher education settings [34,35]. However, SSIs have not yet been applied to clinically relevant difficulties, such as classroom anxiety or FNE, out of the context of mental health–focused trials. No SSIs designed to reduce FNE have been tested among college students. Other trials have found positive outcomes for brief (≤3 sessions) interventions on academic achievement [36-38]. Given the demonstrated utility of brief, scalable interventions in educational environments, there is reason to expect that SSIs may have utility beyond clinical contexts.

### Study Purpose

To address this gap in the literature, we developed and assessed a digital, self-administered SSI designed to reduce FNE and increase confidence in different situations in a classroom setting. In this pilot study, our primary aim was to assess whether the intervention was acceptable and whether it could be feasibly implemented in a large-enrollment college science course taught in an active learning way. The SSI aimed to teach strategies for dealing with FNE in the context of anxiety. We hypothesized that the intervention would impact constructs related to student anxiety such as distress tolerance [39] and intolerance of uncertainty [40] relative to an active control program. We also hypothesized that relative to an active control program, the intervention would increase the intention to persist in science [9,41], which may be threatened by FNE. To our knowledge, this is the first study to develop and test the impact of a digital SSI on college science students with the intent to improve their experiences in the classroom.

## Methods

### Ethical Considerations

Before participant enrollment, all study procedures were approved by Arizona State University’s institutional review board (#STUDY00015263) and preregistered on the Open Science Framework [42]. The trial results have been reported using the CONSORT (Consolidated Standards of Reporting Trials) guidelines [43].

### Recruitment and Procedures

Participants were recruited from a large-enrollment upper-level physiology course taught in an active learning format at Arizona State University, a large public research university located in the southwestern region of the United States. Participant recruitment began on November 11, 2022. The intervention was created as a Qualtrics survey. Qualtrics is a software primarily used for designing and disseminating web-based surveys. The intervention was shared as a Qualtrics link as an assignment with 320 undergraduate students. They were given until November 15, 2022, to complete the survey in exchange for course points (<1%). Alternate assignments were available for students who chose not to participate. Eligibility criteria were (1) comfort speaking and writing in English, (2) consistent access to an internet-equipped device, and (3) aged ≥18 years. Exclusion criteria were (1) responding *yes* to the question “Are you taller than 7 feet?,” which was included as an attention check or (2) responding that they could not commit to completing the entire 45-minute activity.

All participant data were collected and stored using Qualtrics, a secure survey platform. After completing the web-based screener, eligible participants were brought to a page allowing them to document their informed consent, and then directed to a series of preintervention questionnaires. Participants were then randomized (1:1 allocation ratio) to either the intervention condition (*Project Engage*) or an active control condition via a digitally embedded randomizer available in Qualtrics. All participants were blinded to condition assignment, as they were unaware of whether they were in the control group, which was designed to be helpful and contained a positive message, but which did not target the intended target (FNE) of the novel SSI. Participants completed a second set of questionnaires directly after intervention completion to gauge immediate shifts in the outcomes of interest as well as user acceptability.

### Condition Descriptions

**Figure 1:**
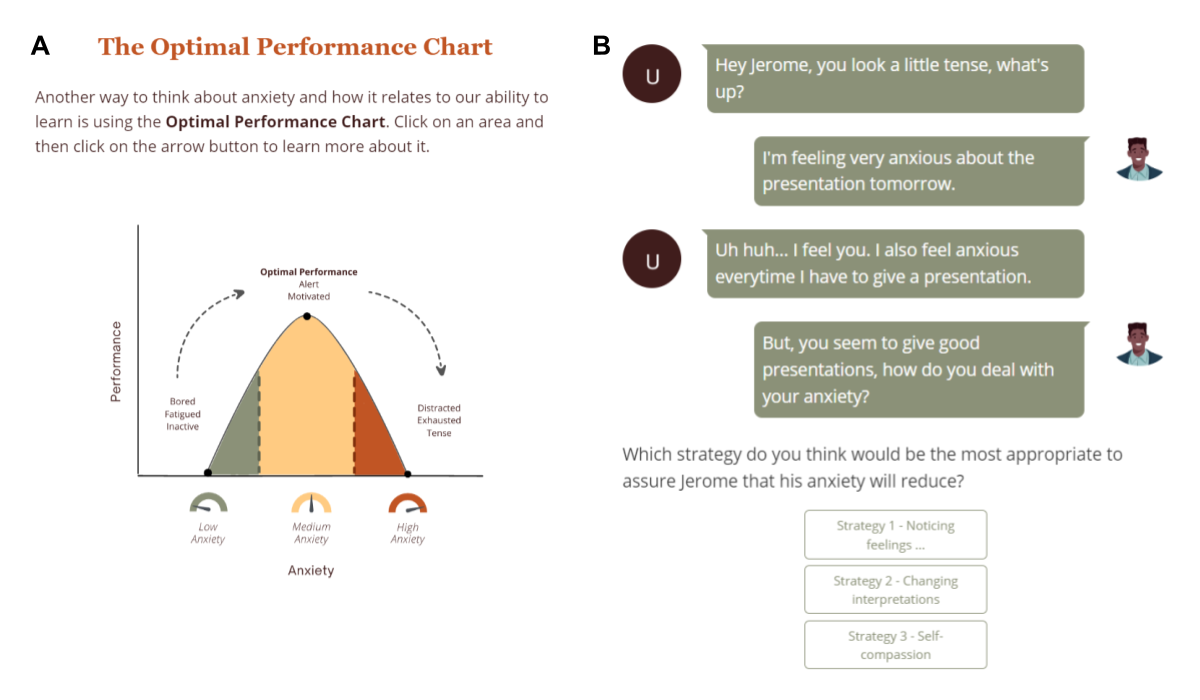
(A) Snapshot of an interactive page in Project Engage. The graph shows how performance varies with anxiety. The participant can click on the colored zones to learn more about how being in that zone of anxiety would affect their performance. (B) Snapshot of an interactive conversation between the participant (left) and a simulated friend (right). Through this conversation, the participant gets a chance to think about which of the techniques they learned in Project Engage would be appropriate in a given situation.

Project Engage [44] is a web-based and self-guided SSI designed to target FNE within the context of an active learning Science, Technology, Engineering, and Medicine (STEM) course. The intervention introduced anxiety in general and discussed coping strategies by providing examples related to FNE in the context of an active learning class. The coping strategies incorporate principles of CBT and mindfulness, encouraging students to reflect on and recognize patterns between their thoughts, feelings, and behaviors in a nonjudgmental manner. Apart from reducing FNE, we expected the intervention to improve student confidence in different social situations in an active learning environment. Project Engage contains 7 main components:

1. Student anxiety within active learning courses, a common outcome of the FNE, is normalized and validated.
2. The potentially adaptive nature of anxiety is described. An
  optimal performance chart
  (which plots anxiety vs performance; Figure 1A) is used to help students differentiate between functional and nonfunctional levels of anxiety.
3. Students are presented with a list of socially evaluative situations that may arise in an active learning classroom and asked which would be most stressful for them (eg, “being involuntarily asked to speak in front of the class”). Then, students identify which feelings, thoughts, and behaviors they may experience in the selected situation.
4. Students are presented with three strategies for managing anxiety in the classroom: (1) noticing physiological responses as anxiety, (2) changing interpretation of anxiogenic situations (ie, cognitive flexibility), and (3) self-compassion.
5. Students read quotes from peers who have successfully used each of the strategies.
6. Students put the strategies into practice by helping a hypothetical peer through a socially evaluative situation via an interactive texting activity (Figure 1B).
7. Students develop an action plan to remind them of how socially stressful situations might make them feel, think, and behave as well as how to handle them.

The main theoretical framework driving the design of Project Engage was the self-determination theory [45]. The self-determination theory posits that it is possible to affect positive behavior change if an intervention supports an individual’s need for competence, relatedness, and autonomy. In line with the principles of self-determination theory and other effective SSIs [24], Project Engage empowers participants to assume an *expert role* (eg, students are experts of their own undergraduate experience) to instill a sense of autonomy. Participants were presented with a set of 3 strategies and a personalized *action plan* to elicit motivation and a sense of competence [26,46,47]. Participants were also presented with testimonials from peers to give them a sense of relatedness to others. To the best of our knowledge, Project Engage is the first intervention based on self-determination theory aiming to increase student confidence in the context of higher education.

The control condition, which contained a generally positive message linked to well-being and mental health, was similarly web-based and self-guided to account for any nonspecific outcomes of completing an web-based activity. Participants also began this activity by identifying a socially evaluative situation that would elicit anxiety and then choosing the thoughts, feelings, and actions that they would associate with that situation. Instead of being provided with evidence-based strategies, the participants in the control condition were presented with a list of resources specific to their university. Similar to Project Engage, the control condition incorporated open-ended prompts to encourage a similar level of participant effort and engagement.

### Measures

#### Demographics

The following demographic information was collected: gender identity, racial and ethnic identity, sexual orientation, LGBTQ+ (lesbian, gay, bisexual, transgender, queer, and others) identity, parental education level, financial stability, household income, primary language spoken, grade point average, international student status, and disability status.

##### Assessing FNE

The straightforward Brief Fear of Negative Evaluation (BFNE-S) subscale [12] was used to assess FNE. Only positively worded items (ie, assessing the presence, rather than absence, of fear) were presented. Students were asked to rate 8 statements (eg, “I am usually worried about what kind of impression I make”) on a 5-point Likert scale (1=“Not at all characteristic of me” and 5=“Extremely characteristic of me”) specifically within the context of a STEM active learning course. The total scores range from 8 to 40, with higher scores indicating greater fear of negative social evaluation. As this measure asks students to reflect on their past experiences in active learning courses, BFNE-S scores were not expected to change before intervention to immediately after the intervention. Therefore, the BFNE-S was only presented before the intervention. The BFNE-S has been found to have sound psychometric properties, including internal consistency, convergent validity, and divergent validity, in adults with social anxiety disorder [12].

##### Program Acceptability

The acceptability and feasibility of the program were assessed using the Program Feedback Scale (PFS) [48]. The PFS contains 7 items (eg, “I enjoyed the activity” and “I agree with the activity’s message”), which students rated on a 5-point Likert scale with response anchors at –2 (“Really disagree”) and 2 (“Really agree”). As per the study preregistration, an average score of ≥0.5 indicates adequate acceptability. This scale was specifically developed for use in brief digital interventions [48].

#### Proximal Outcomes

##### Overview

Changes in distress tolerance, intolerance of uncertainty, confidence, and intention to persist in science were evaluated as preintervention to immediate postintervention outcomes. Importantly, we are assessing immediate shifts in the SSI targets because immediate shifts may predict more positive long-term changes in outcomes related to well-being and mental health [49]. Therefore, the following measures were used to better understand the potential effectiveness and utility of the intervention.

##### Distress Tolerance

The Distress Tolerance Scale (DTS) [50] was used to assess distress tolerance. Students rated 16 statements (eg, “I can’t handle feeling distressed or upset”) on a 5-point Likert scale (1=“Strongly disagree at this moment” and 5=“Strongly agree at this moment”; we reverse-coded the scores during analysis to be congruent with the original scoring scheme) specifically within the context of a STEM active learning course. This measure was modified to assess levels of *in-the-moment* distress tolerance by asking students to consider how much each item described them *at this moment*. Total scores range from 16 to 80, with higher scores indicating higher levels of distress tolerance. This measure was presented both before and immediately after the intervention. The DTS has shown acceptable internal consistency and construct validity in previous student samples [50].

##### Intolerance of Uncertainty

The Intolerance of Uncertainty Scale-12 (IUS-12) [51] was used to assess intolerance of uncertainty. Students rated 12 statements (eg, “I can’t stand being taken by surprise”) on a 5 point Likert scale (1=“Not at all like me at this moment” and 5=“Entirely like me at this moment”) specifically within the context of a STEM active learning course. This measure was modified to assess levels of *in-the-moment* intolerance of uncertainty by asking students to consider how much each item described them *at this moment*. Total scores range from 12 to 60, with higher scores indicating higher levels of intolerance of uncertainty. This measure was presented both before and immediately after the intervention. The IUS-12 has shown high levels of internal consistency in undergraduate samples [51].

##### Confidence

We aimed to assess students’ confidence in contributing to discussions in different contexts: one-on-one conversations with the instructor, in small groups, and with the whole class. Despite a thorough literature search, we were unable to find suitable, previously developed, and validated measures to assess students’ confidence in contributing to these unique contexts. As a result, confidence was assessed by asking students how confident they would be to complete a socially evaluative activity (eg, “Answer a question”) within 3 different environments (small group discussion, whole class discussion, and one-on-one discussion with the instructor). For each environment, students completed 4 items rating their *in-the-moment* comfort levels for completing an activity in a large-enrollment college science course (1=“Not at all confident at this moment” and 5=“Very confident at this moment”; Multimedia Appendix 1 provides full questionnaire). Scores for each environment ranged from 4 to 20, with higher scores indicating higher confidence levels within that environment. These scales were developed in collaboration with a psychometrician who was also on the advisory board for this study. To establish cognitive validity, we conducted 6 think-aloud interviews with undergraduate science students, iteratively revising after each think-aloud to ensure that the questions were being interpreted as intended [52]. These scales were piloted with a sample of undergraduate science students (N=566). Factor analyses indicated excellent model fit (small group discussion Comparative Fit Index (CFI)=1.00, whole class discussion CFI=0.99, and one-on-one discussion with instructor CFI=0.98), and each of the 3 scales was negatively correlated with FNE, as would be expected. The McDonald’s omega values indicated adequate internal consistency (all>0.9). Confidence scales for each environment were presented both before and immediately after the intervention.

##### Intention to Persist in Science

Students were asked to answer the question “To what extent do you intend to pursue a science-related research career?” at this moment on an 11-point Likert scale (0=“Definitely will not” and 10=“Definitely will”). This item was presented both before and immediately after the intervention. This item is hypothesized to be a proximal predictor of long-term retention in the sciences for underrepresented science students [53].

### Analytic Plan

#### Overview

All demographic variables were reported as percentages of students who endorsed each response option. Completion rates were calculated for each condition as the percentage of students within that condition who completed the entire survey. Mean baseline levels of all primary outcome variables, as well as BFNE-S scores, were reported. Independent 2-tailed *t* tests were performed to check for differences in the mean baseline levels of each affective variable. For all analyses, the effects were considered significant if *P*<.05.

#### Primary Outcomes

##### Affective Outcomes

Multiple linear regression models were used to evaluate the effect of condition assignment, separately, on each primary outcome: distress tolerance, intolerance of uncertainty, confidence, and intention to persist in science. In each model, the baseline level of the affective outcome and the intervention condition (experimental=1, control=0) were included as a predictor. As per the preregistered cutoff, the effect of the conditions was considered significant if *P* was <.05.

##### Program Acceptability

For both conditions, mean scores were calculated. For experimental group participants, all scores have been reported for each item on the PFS. For the control group participants, we have reported the mean acceptability score across the 7 items.

#### Secondary Outcomes

##### Student Performance

Student performance was indexed by students’ scores on the first 3 examinations. As the first 2 examinations took place before randomization, the first 2 examination averages and condition assignments were added as predictors to the regression model, which predicted the third examination score. A *P* value of <.05 for condition assignment was preregistered as a significant differential effect in this model.

##### Qualitative Responses

Qualitative feedback was collected about students’ experiences with the digital intervention, as well as the strategies they currently use to handle anxiety in interactive classrooms. Responses were thematically analyzed using practices delineated by Braun and Clarke [54]. An inductive approach was used to identify and report commonly recurring themes in the data.

### Missing Data and Correcting for Multiple Tests

List-wise deletion was used to exclude noncomplete responses (ie, participants who dropped out of either condition). No missing data were imputed. During the thematic analysis of qualitative data, participants were excluded if their responses made it apparent that they were non-English speakers. In cases where participants submitted multiple responses, their more complete response was used for the analyses. If both responses were equally complete, their initial response was used. Preregistered multiple regressions were corrected using the Benjamini-Hochberg method [55].

### Software Packages

We used R (version 4.2.2; R Foundation for Statistical Computing [56]) for running statistical analyses and the MOTE package to calculate effect sizes [57].

### Sample Size

As this was a pilot study, we did not perform a sample size calculation. However, we anticipated that 320 participants would be a large enough sample to assess the feasibility and acceptability of the intervention and to provide preliminary evidence of efficacy.

## Results

### Participant Characteristics

**Figure 2:**
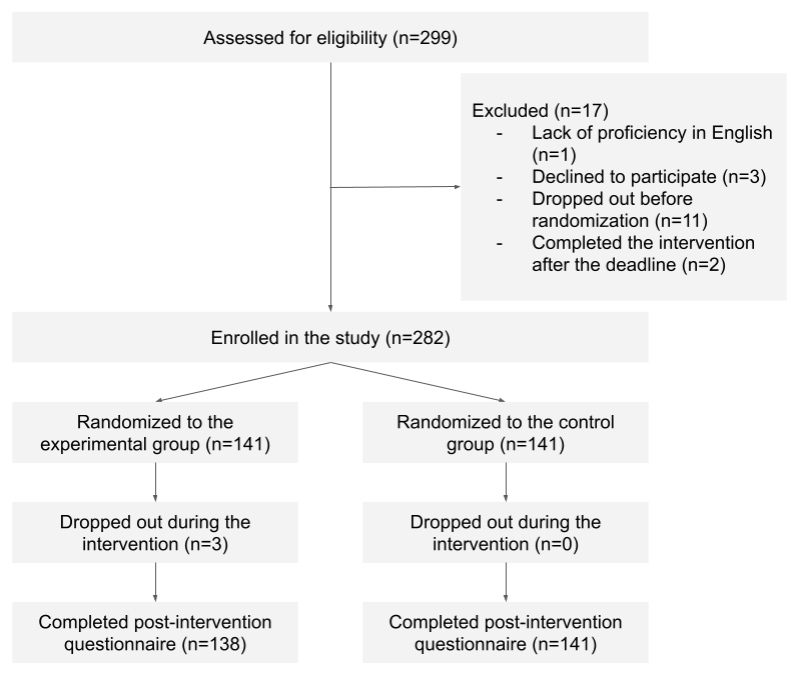
The flow of participants in the pilot trial.

Of the 320 students who were invited to participate in the study, 299 (93.4%) students started the survey. Of these 299 participants, 282 (94.3%) met our inclusion criteria and were randomly assigned to the experimental (n=141, 50%) or control group (n=141, 50%; Figure 2). A total of 138 participants in the experimental group and 141 participants in the control group completed the postintervention questionnaires. The participants in both groups were found to be balanced in terms of demographic factors (Table 1) and baseline scores on the BFNE-S (*P*=.59), IUS-12 (*P*=.21), persistence scale (*P*=.27), and confidence scale (*P*=.77). Only for DTS, the baseline score of experimental group participants was significantly higher than that of the control group participants (*P*=.02).

**Table 1:** Demographic characteristics of participants recruited in the study.

Group		Experimental (n=141)	Control (n=141)	All (n=282)
Age (y), mean (SD)		20.78 (1.89)	21.52 (3.23)	21.15 (2.67)
**Gender identity, n (%)**				
	Men	50 (35.5)	49 (34.7)	99 (35.1)
	Women	89 (63.1)	89 (63.1)	178 (63.1)
	Nonbinary	2 (1.4)	2 (1.4)	4 (1.4)
	Another gender	—^a^	1 (0.7)	1 (0.3)
**Race or ethnicity^b^, n (%)**				
	American Indian or Alaska Native	4 (2.8)	3 (2.1)	7 (2.5)
	Asian (including South Asian)	43 (30.5)	34 (24.1)	77 (27.3)
	Black or African American	10 (7.1)	8 (5.7)	18 (6.4)
	Hispanic or Latinx	25 (17.7)	31 (22)	26 (19.9)
	Native Hawaiian or another Pacific Islander	2 (1.4)	2 (1.4)	4 (1.4)
	White (non-Hispanic; includes Middle Eastern)	69 (48.9)	79 (56)	148 (52.5)
	Prefer not to answer	2 (1.4)	2 (1.4)	4 (1.4)
	Other (specify)	1 (0.7)	2 (1.4)	3 (1.1)
**LGBTQ+^c^ status, n (%)**				
	Yes	27 (19.1)	26 (18.4)	53 (18.8)
	No	112 (79.4)	115 (81.6)	227 (80.5)
	Did not answer	2 (1.4)	—	2 (0.7)
**Gender identity among participants who identify as LGBTQ+^b^, n (%)**				
	Transgender	1 (0.7)	1 (0.7)	2 (0.7)
	Gender nonbinary	5 (3.5)	0 (0)	5 (1.8)
	Gender queer	0 (0)	3 (2.1)	3 (1.1)
	Third gender	0 (0)	0 (0)	0 (0)
	Two-spirited	0 (0)	0 (0)	0 (0)
	Agender	1 (0.7)	1 (0.7)	2 (0.7)
	A gender not listed	17 (12.1)	14 (9.9)	31 (10.9)
**Sexual orientation of participants who identify as LGBTQ+^b^, n (%)**				
	Asexual	0 (0)	2 (1.4)	2 (0.7)
	Lesbian or gay	12 (8.5)	6 (4.2)	18 (6.4)
	Bisexual	13 (9.2)	18 (12.8)	31 (10.99)
	Queer	7 (4.9)	2 (1.4)	9 (3.2)
	Questioning	3 (2.1)	2 (1.4)	5 (1.8)
	An identity not listed	3 (2.1)	0 (0)	3 (1.1)
**Parental educated, n (%)**				
	Did not complete high school	7 (4.9)	10 (7.1)	17 (6)
	High-school diploma or GED^d^	17 (12.1)	14 (9.9)	31 (10.9)
	Some college but no degree	17 (12.1)	12 (8.5)	29 (10.28)
	Associate degree (eg, AA^e^ and AS^f^)	5 (3.5)	9 (6.4)	14 (4.9)
	Bachelor’s degree (eg, BA^g^ and BS^h^)	38 (26.9)	38 (26.9)	76 (26.9)
	Master’s degree (eg, MA^i^, MS^j^, MEd^k^, MSW^l^, MBA^m^)	32 (22.7)	32 (22.7)	64 (22.7)
	Higher than a master’s degree (eg, PhD^n^, MD^o^, JD^p^)	24 (17)	24 (17)	48 (17)
	Prefer not to answer	1 (0.7)	1 (0.7)	2 (0.7)
	Other	—	1 (0.7)	1 (0.3)
**Financial stability, n (%)**				
	Yes	82 (58.2)	83 (58.9)	165 (58.5)
	Yes, but only sometimes	40 (28.4)	37 (26.2)	77 (27.3)
	No	13 (11.3)	19 (13.5)	35 (12.4)
	Prefer to answer	3 (2.1)	2 (1.4)	5 (1.8)
**Household income (US $), n (%)**				
	Low income (<25,000)	10 (7.1)	6 (4.3)	16 (5.7)
	Middle-low income (25,000-49,999)	16 (11.3)	29 (20.6)	45 (15.9)
	Middle income (50,000-99,999)	32 (22.7)	40 (28.4)	72 (25.5)
	Middle-high income (100,000-199,999)	50 (35.5)	37 (26.2)	87 (30.9)
	High income (≥200,000)	25 (17.7)	18 (12.8)	43 (15.2)
	Prefer not to answer	8 (5.7)	11 (7.8)	19 (6.7)
**Language, n (%)**				
	English	135 (95.7)	123 (87.2)	258 (91.5)
	Spanish	4 (2.8)	7 (4.9)	11 (3.9)
	Other	2 (1.4)	11 (7.8)	13 (4.6)
GPA^q^, mean (SD)		3.62 (0.4)	3.62 (0.38)	3.62 (0.39)
**International student status, n (%)**				
	Yes	4 (2.8)	4 (2.8)	8 (2.8)
	No	137 (97.2)	137 (97.2)	274 (97.2)
**Disability, n (%)**				
	Yes	19 (13.5)	15 (10.6)	34 (12.1)
	No	122 (86.5)	126 (89.4)	248 (87.9)

^a^Not available.

^b^Participants could select more than 1 option.

^c^LGBTQ+: lesbian, gay, bisexual, transgender, queer, and others.

^d^GED: General Educational Development.

^e^AA: Associate of Arts.

^f^AS: Associate of Science.

^g^BA: Bachelor of Arts.

^h^BS: Bachelor of Science.

^i^MA: Master of Arts.

^j^MS: Master of Science.

^k^MEd: Master of Education.

^l^MSW: Master of Social Work.

^m^MBA: Master of Business Administration.

^n^PhD: Doctorate of Philosophy.

^o^MD: Doctor of Medicine.

^p^JD: Juris Doctor.

^q^GPA: Grade Point Average.

### Acceptability, Feasibility, and Length

**Figure 3:**
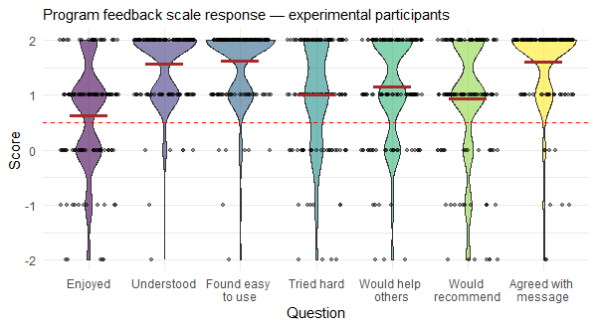
Violin plot showing experimental group participants’ responses on the Program Feedback Scale. Each violin corresponds to a question in the Program Feedback Scale. Black dots indicate user responses. Solid horizontal red lines indicate the mean values and the dotted red line indicates the acceptability cut-off score of 0.5.

A total of 137 participants in the experimental group submitted the PFS. Overall, Project Engage scored above our acceptability criteria of 0.50 on the PFS (M*_exp_*=1.22). The mean acceptability score of Project Engage was higher than that of the control version on the same scale (M*_con_*=1.07). All individual items on the PFS received a feedback score higher than the acceptability cutoff (Figure 3). Among the different aspects of the intervention, the question on whether the activity was easy to use received the highest rating, and the question on whether the activity was enjoyable received the lowest rating. According to the text-based feedback provided by the participants on the PFS, the positive aspects of Project Engage were that (1) the techniques will be useful for dealing with anxiety, (2) it was engaging, and (3) it was easy to use. Most participants reported that they would not change anything about the intervention. Apart from that, the most common negative feedback was that Project Engage was too long. Only one participant reported that the intervention affected them negatively; they mentioned, “I am currently going through a very hard time and I felt like this activity just brought more of those emotions to the surface.”

The completion rate of the overall study, including the baseline questionnaires, the intervention, and the postintervention questionnaires, was 95.22%. After randomization, only 3 participants dropped out from the experimental group, and 0 participants dropped out from the control group. For the participants randomized to the experimental and control groups, the completion rates were 97.87% and 100%, respectively.

**Figure 4:**
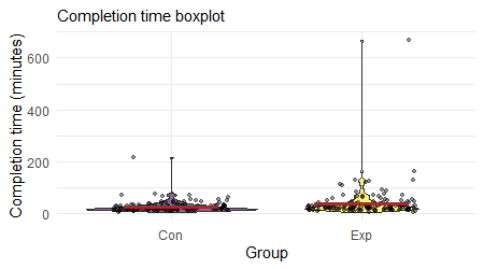
Violin plot showing the total time spent by the Control and Experimental group participants with Project Engage. Outliers indicate participants who completed the survey but did not close the browser tab.

On average, participants in the experimental and control groups spent 39.98 minutes and 26.68 minutes in the overall survey, respectively (Figure 4). Figure 4 shows that a few users have spent an unusually high amount of time with the intervention. These outliers were most likely participants who did not close the survey tab on their browser once they completed the survey. Therefore, to obtain a better estimate of the time spent by the participants, we calculated the total time spent by the participants on the individual pages of the survey and took the sum of those times to calculate the total time spent. We had these data for most but not all pages, so it is a slight underestimation of the actual amount of time spent on the survey. Using this method, we found that the average time spent by the experimental and control group participants was 27.08 minutes and 19.53 minutes, respectively.

### Effects on Participants' Confidence

We ran multiple linear regressions with baseline confidence scores and intervention groups as predictors (Table 2 provides full regression results). The experimental group participants showed a significant increase in *in-the-moment* confidence compared with the control group participants for speaking in small groups (1.07 vs 0.08; *P_adj_*=.01; Cohen *d*=0.34, 95% CI 0.10-0.58) and in the whole class (1.57 vs 0.12; *P_adj_*<.001; Cohen *d*=0.51, 95% CI 0.26-0.74), but not for a one-on-one with the instructor (0.79 vs 0.13; *P_adj_*=.05; Cohen *d*=0.27, 95% CI 0.03-0.51). Participants in the experimental group also reported significant increases in total confidence— calculated as the sum of the 3 subscales—compared with the control group (3.44 vs 0.34; *P_adj_*<.001; Cohen *d*=0.48, 95% CI 0.24-0.72). There was a significant difference between baseline total confidence scores between the experimental group participants whose (1) parents had attended or completed school, (2) parents had an associate degree, bachelor’s degree, or had attended college but had no degree, and (3) parents had a master’s or higher degree (*F*_2,134_=4.59; *P*=.01). After the intervention, there was no significant difference between the total confidence in the 3 groups (*F*_2, 133_=2.17, *P*=.12). We divided the experimental group into subgroups based on LGBTQ+ status (yes or no), disability status (yes or no), primary language (English or non-English), family income (low income, middle income, or high income), and parental education (first-generation college students or continuing-generation college students). We did not find significant differences in the baseline confidence levels of these subgroups.

**Table 2:** Results of multiple linear regression models predicting intervention effects on confidence scores.

		β (SE)	*P* value
**Confidence in small group**			
	Intercept	3.24 (.65)	<.001
	Condition	1.01 (.35)	.01^a^
	Baseline score	.77 (.04)	<.001
**Confidence in the whole class**			
	Intercept	1.69 (.42)	<.001
	Condition	1.42 (.34)	<.001^a^
	Baseline score	.82 (.04)	<.001
**Confidence in one-on-one with the instructor**			
	Intercept	3.63 (.63)	<.001
	Condition	.78 (.35)	.05^a^
	Baseline score	.76 (.04)	<.001
**Overall confidence**			
	Intercept	5.82 (1.57)	<.001
	Condition	3.22 (.81)	<.001^a^
	Baseline score	.85 (.04)	<.001

^a^*P* values have been adjusted using the Benjamini-Hochberg method.

### Effects on DTS, IUS-12, and Intention to Persist in a Science-Related Career

Table 3 reports the baseline and postintervention scores (if available) in the different questionnaires administered to the participants. The changes in the scores were not statistically significant on DTS, IUS-12, or the intention to persist in science scale.

**Table 3:** Baseline and postintervention scores of participants recruited in the study.

Assessment scale			Time point, mean (SD)												
			Baseline					Postintervention					Postintervention—baseline change^a^		
			Experimental group		Control group		Experimental group			Control group		Experimental group			Control group
Brief Fear of Negative Evaluation-S			22.96 (8.73)		22.39 (9.34)		N/A^b^			N/A		N/A			N/A
Distress Tolerance Scale			56.69 (12.71)		60.07 (11.52)		58.01 (13.67)			60.36 (12.54)		1.32			0.29
Intolerance of Uncertainty Scale-12			31.49 (9.75)		29.99 (10.06)		29.79 (10.6)			29.33 (10.98)		–1.7			–0.66
**Confidence**															
	Small group	14.04 (4.22)		14.21 (4.35)		15.11 (4.31)			14.29 (4.55)		1.07			0.08	
	Whole class	8.39 (4.34)		8.76 (4.40)		9.96 (4.63)			8.88 (4.45)		1.57			0.12	
	One-on-one	15.14 (4.21)		14.94 (4.87)		15.93 (4.23)			15.07 (4.71)		0.79			0.13	
	Total	37.55 (10.38)		37.91 (10.79)		40.99 (10.97)			38.25 (11.34)		3.44			0.34	
Intention to persist in science			6.46 (2.78)		6.08 (2.99)		6.33 (2.85)			6.29 (2.94)		–0.13			0.21

^a^These values represent the change in the mean values of experimental and control group participants from baseline to postintervention. Hence, SD values are not available.

^b^N/A: not applicable (BFNE-S was not administered at postintervention).

### Exploratory Analysis

#### Effects on Student Performance

Student performance data were available as 3 examination points (examination 1, examination 2, and final examination), participation points, and homework points. Out of these, examination 1 and examination 2 were conducted—before the intervention, and the final examination was conducted—after the intervention. We ran a multiple regression with intervention conditions (experimental=1, control=0) and an average of examination 1 percentage and examination 2 percentage as the predictor variables and final examination percentage as the predicted variable. We did not find any effects of the intervention condition on the final examination percentage (*P*=.96). We did not find any within-group effects on the examination performance for experimental (*P*=.15) or control group participants (*P*=.27). For participation and homework points, preintervention versus postintervention data were not available; therefore, no analysis was performed.

#### Analysis of Text-Based Responses for Experimental and Control Group Participants

The experimental and control group participants were asked to select the most anxiety-inducing situation in a class. The most commonly endorsed anxiety-inducing situation was *“*Being involuntarily asked to speak in front of the class*”* (Table 4). The most common feelings, thoughts, and behaviors were *“*Heart racing*,”* *“*I shouldn’t be this stressed out,” and *“*Overthink responses,” respectively. The experimental group participants were presented with 3 strategies (*noticing physiological feelings and trusting that they will reduce*, *changing interpretations*, and *practicing self-compassion*) to deal with the FNE. As a last step of Project Engage, we asked the experimental group participants to select a strategy that they would use to deal with anxiety. The most commonly selected strategy was *noticing physiological feelings and trusting that they will reduce.*

**Table 4:** Anxiety-inducing situations, and related feelings, thoughts, and behaviors selected by participants (N=282)^a^.

			Selected by, n (%)
**Situations**			
	Being involuntarily asked to speak in front of the class	151 (53.55)	
	Presenting in front of the class individually	95 (33.69)	
	Asking for extra help	11 (3.9)	
	Asking a question in front of everyone	10 (3.55)	
	Voluntarily answering a question in front of the class	4 (1.42)	
	Talking with the instructor one-on-one	4 (1.42)	
	Presenting in a group presentation	4 (1.42)	
	Participating in small group discussion	1 (0.35)	
**Feelings**			
	Heart racing	258 (91.49)	
	Sweaty palms	180 (63.83)	
	Trembling	156 (55.32)	
	Short, shallow breathing	115 (40.78)	
	Upset stomach	110 (39)	
	Headaches	61 (21.63)	
	Muscle aches	23 (8.16)	
**Thoughts**			
	I should not be this stressed out	184 (65.25)	
	Everyone will think I am dumb	182 (64.54)	
	The professor will think I am stupid	139 (49.29)	
	Other people can handle this situation—what’s wrong with me?	134 (47.52)	
	Other students will laugh at me	108 (38.29)	
	I cannot handle this	103 (36.52)	
	People will make jokes about me if I get the wrong answer	89 (31.56)	
	The professor will be disappointed in me	73 (25.89)	
	This is the worst possible thing that could happen to me	68 (24.11)	
	Other students would not want to work with me	57 (20.21)	
	I am never going to succeed	48 (17.02)	
**Behaviors**			
	Overthink responses	202 (71.63)	
	Avoid eye contact	158 (56.03)	
	Prepare more	150 (53.19)	
	Struggle to think through things	127 (45.04)	
	Participate less in class	98 (34.75)	
	Try to get away from the stressful situation	97 (34.39)	
	Avoid talking	71 (25.18)	
	Consider dropping the class	37 (13.12)	

^a^The numbers indicate how many participants selected each situation, feeling, thought, and behavior. Participants could select only one situation but multiple feelings, thoughts, and behaviors.

## Discussion

### Principal Findings

We have presented the results of a pilot trial evaluating a self-administered digital SSI designed to help students deal with the FNE in social-evaluative situations within the context of an active learning class. Results from the pilot trial conducted with 282 participants showed that the intervention was acceptable to the students and feasible for implementation in a large-enrollment classroom setting. In addition, the results showed that the experimental intervention improved *in-the-moment* confidence compared with the control intervention.

Overall, the SSI was acceptable to the participants, with most aspects receiving an average rating of >1 on a scale of –2 to +2. The aspect of whether the activity was enjoyable received a lower rating compared with the other aspects of the SSI. The intervention was developed and provided using Qualtrics. As Qualtrics is primarily a software designed to administer web-based surveys, it provides limited opportunities to create an interactive intervention, which might have contributed to the relatively low rating on enjoyability. In the future, bespoke software based on Project Engage might provide more opportunities to include interactive features and make the intervention more enjoyable. A bespoke software would also allow added opportunities to send reminders to the participants based on the strategies they have learned in the SSI.

The overall survey had a very high completion rate (>95%) compared with open trials of self-administered digital SSIs [29]. This indicates that the intervention is feasible for implementation and evaluation via a large-scale randomized controlled trial (RCT). Although we cannot determine the exact reason based on the available data, the high completion rate may be because of multiple factors: (1) acceptability and relevance of the intervention to the participant population, (2) being recommended by a trusted source (a course instructor in this case), (3) being offered as an assignment, and (4) being offered course points (although <1% of the total course points) for completing the survey. This finding has implications for the real-world dissemination of SSIs and other mental health interventions in university settings. Although being offered coupons or monetary benefits would be difficult to scale up in the real world, large-scale dissemination of interventions as assignments in college courses and as a way to earn course points may be relatively more feasible.

Compared with the control group, Project Engage was able to increase *in-the-moment* confidence immediately after the intervention among participants, with a medium effect size (Cohen *d*=0.48). The intervention also led to increased *in-the-moment* confidence for speaking in small groups and in the whole class. The corresponding effect sizes were medium (Cohen *d*=0.34) and large (Cohen *d*=0.51), respectively. Our results are comparable with the short-term (0-2 weeks) postintervention effects of other SSIs (Hedges *g*=0.46) [25].

The increase in confidence for speaking one-on-one with the instructor was not statistically significant. One potential explanation is that baseline confidence for speaking one-on-one with the instructor was high and therefore, there was less scope for improvement. Indeed, we found significant differences in the 3 subscales of confidence (small group discussion, whole class discussion, and one-on-one discussion with instructor) at baseline for the experimental group (*F*_2, 415_=100.3, *P*<.001). Post hoc analyses using pairwise *t* tests and Benjamin-Hochberg correction revealed that baseline confidence was significantly lower for speaking in small groups (*P*=.03) and the whole class (*P*<.001) as compared with speaking one-on-one with the instructor.

In the pilot study, we did not observe an effect of Project Engage on *in-the-moment* scores on the DTS, the IUS-12, or the Intention to Persist in Science scale. Although we cannot know why the results were null, distress tolerance, intolerance of uncertainty, and the choice of a future career are stable attributes that may be unlikely to change within a short period of 30-40 minutes. In future RCTs, long-term follow-ups are required to check whether the intervention has a delayed effect on these attributes.

### Comparison With Prior Work

Upon reviewing the literature, we were unable to find other digital SSIs aiming to improve student confidence in the context of higher education. However, other studies have evaluated single-session treatments targeting FNE and related constructs [58-60]. Hindo and González-Prendes [58] evaluated an SSI that gradually exposed participants from least to most anxiety-inducing situations in a group setting. The total intervention duration was 3 hours long and they found that the participants had reduced social anxiety and public speaking anxiety immediately after the intervention with a large effect size [58]. However, this study lacked a control group, and hence, the effect size might be inflated. Another study by Knutsson et al [59] evaluated a 90-minute therapist-led SSI delivering exposure or imagery rescripting-based treatments. They found that both treatments reduced FNE and that the gains were maintained at 4-week follow-up. Stefan et al [60] evaluated a 2 hours 30 minutes long web-based group session providing contextual schema therapy. This intervention reduced FNE from pre- to posttest compared with a waitlist control. Overall, these studies provide evidence that single-session treatments can help with the FNE and related constructs. Compared with these therapist-led interventions, Project Engage, being fully self-administered and digital, has a higher scalability.

### Strengths and Limitations

Strengths of this study include its large sample size and low dropout rate. A limitation of this study was the lack of long-term follow-up data. Although Project Engage resulted in *in-the-moment* improvements on some measures compared with the active control group, it is unclear whether these improvements were sustained over time. This study did not collect such data as it aimed primarily to test the feasibility and acceptability of Project Engage. However, the promising results of this pilot trial suggest that collecting long-term follow-up data may be a future direction worth pursuing.

Another limitation of this study was the discrepancy between the experimental group and the control group regarding the time spent on the intervention. The experimental group participants spent significantly more time with the intervention compared with the control group participants. Although the activities were intended to be approximately time matched, this discrepancy may be because of the type of information presented in each activity. It is possible that the content presented in Project Engage provided more opportunities for reflection and deeper processing than the control condition, depending on perceived personal relevance and familiarity of the information to each student. In future studies, the active control condition may be altered to ensure that the time spent on the activity is comparable with that of the experimental intervention.

Given the measures of interest in this study—FNE, confidence in the classroom, distress tolerance, and intolerance of uncertainty—we had to rely on self-report scales, which are susceptible to common method bias.

Finally, although all participants were recruited from one upper-level physiology course at Arizona State University, they had diverse demographic backgrounds (Table 1). Consequently, our results might generalize to student populations at other US universities, but their generalizability beyond the US might be limited.

### Future Directions

As college science courses continue to transition from traditional lecture format to active learning, bolstering student confidence as it relates to speaking in class is an important step in reducing FNE and improving overall student experiences [8]. This study demonstrates that the theoretical basis and scalability of clinical psychology interventions can be leveraged to improve student confidence at scale. There is substantial evidence that women have higher FNE compared with men [14,61,62]. In addition, nonbinary individuals, persons excluded because of their ethnicity or race, first-generation college students, LGBTQ+ students, and disabled students have also been found to express disproportionately high levels of FNE in the context of college science courses compared with their respective peer groups [14]. These differences in FNE likely help explain why women’s voices are underrepresented in both whole-class and small-group discussions in college biology courses [63-66] and why there are suspected participation gaps among other underrepresented groups [67]. Therefore, it is imperative to test whether equipping students with the skills to cope with FNE may disproportionately benefit groups that are reluctant to participate in class for these reasons. Future studies can investigate whether Project Engage differentially affects marginalized individuals and whether the actual participation of students in these groups changes after being exposed to the intervention. Researchers hypothesize that enhanced participation may lead to additional long-term outcomes, including better performance [68,69], college retention rates, and decisions to pursue a career or leadership position in academic science [70,71]. As such, we view Project Engage as a critical step in working to create a more diverse and inclusive scientific community.

### Conclusions

Active learning classes, although effective at improving students’ learning, may exacerbate evaluation-related anxiety owing to the structure of coursework and learning activities, creating a need for interventions that support students in succeeding in these learning environments. Researchers from Science Education and Clinical Psychology worked together to create Project Engage, an intervention designed to provide psychoeducation and teach coping skills to students who experience FNE within active learning classes. In a pilot RCT, Project Engage had a high completion rate and received a high acceptability rating. These results demonstrate that a digital, self-administered SSI can be feasibly implemented in large classroom settings. In addition, participants randomized to complete Project Engage reported greater increases in overall confidence in engaging in small group discussions and whole class discussions, from baseline to immediately after intervention, compared with participants in an active control condition. Fully powered RCTs investigating Project Engage with a longer follow-up period are warranted to determine whether these effects are sustained over time. Such an investigation may provide stronger evidence regarding the efficacy of Project Engage in improving the classroom engagement and coping skills of students in active learning classes.

## Appendix

Multimedia Appendix 1 The full questionnaire developed for the study.

Multimedia Appendix 2 CONSORT-eHEALTH checklist (V 1.6.1).

## Abbreviations

BFNE-S: Brief Fear of Negative Evaluation
CBT: cognitive behavioral therapy
CFI: Comparative Fit Index
CONSORT: Consolidated Standards of Reporting Trials
DTS: Distress Tolerance Scale
FNE: fear of negative evaluation
IUS-12: Intolerance of Uncertainty Scale-12
LGBTQ+: lesbian, gay, bisexual, transgender, queer, and others
PFS: Program Feedback Scale
RCT: randomized controlled trial
SSI: single-session intervention
STEM: Science, Technology, Engineering, and Medicine

## Data Availability

Deidentified data may be available on reasonable request.
