# Supplementary material for: A Digital Single-Session Intervention (Project Engage) to Address Fear of Negative Evaluation Among College Students: Pilot Randomized Controlled Trial
Source: JMIR Ment Health. 2023 Nov 23;10:e48926. doi: 10.2196/48926 (PMC10704327; doi:10.2196/48926)
Supplement: Multimedia Appendix 1 [file mental_v10i1e48926_app1.docx]

**A digital single-session intervention (Project Engage) to address fear of negative evaluation among college students: a pilot randomized controlled trial**

Arka Ghosh, Katherine A. Cohen, Laura Jans, Carly A. Busch, Riley McDanal, Yuanyuan Yang, Katelyn M. Cooper, and Jessica L. Schleider

**Confidence scale**

**Confidence scale**

At this moment, how confident would you be to complete any of the following in a ***small group discussion*** of 3-5 people in a large-enrollment college science course? Options range from Not at all confident at this moment (1) to Very confident at this moment (5).

|  | Not at all | Slightly | Somewhat | Moderately | Very |
| --- | --- | --- | --- | --- | --- |
| Answer a question | 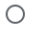 | 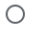 | 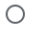 | 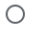 | 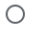 |
| Ask a question | 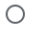 | 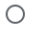 | 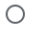 | 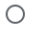 | 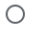 |
| Contribute a comment | 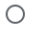 | 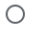 | 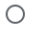 | 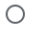 | 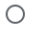 |
| Contribute a response you are unsure of | 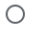 | 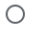 | 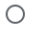 | 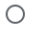 | 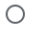 |

At this moment, how confident would you be to complete any of the following in a ***whole class discussion*** in a large-enrollment college science course? Options range from Not at all confident at this moment (1) to Very confident at this moment (5).

|  | Not at all | Slightly | Somewhat | Moderately | Very |
| --- | --- | --- | --- | --- | --- |
| Answer a question | 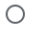 | 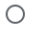 | 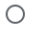 | 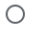 | 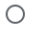 |
| Ask a question | 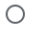 | 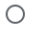 | 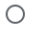 | 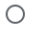 | 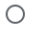 |
| Contribute a comment | 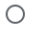 | 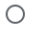 | 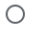 | 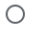 | 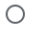 |
| Contribute a response you are unsure of | 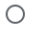 | 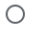 | 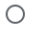 | 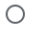 | 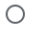 |

At this moment, how confident would you be to complete any of the following in a **one-on-one discussion with the instructor** in a large-enrollment college science course? Options range from Not at all confident at this moment (1) to Very confident at this moment (5).

|  | Not at all | Slightly | Somewhat | Moderately | Very |
| --- | --- | --- | --- | --- | --- |
| Answer a question | 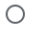 | 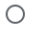 | 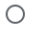 | 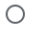 | 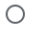 |
| Ask a question | 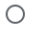 | 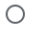 | 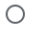 | 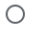 | 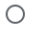 |
| Contribute a comment | 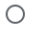 | 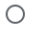 | 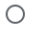 | 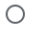 | 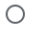 |
| Contribute a response you are unsure of | 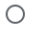 | 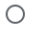 | 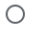 | 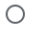 | 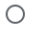 |
